# Phylogenetic Inconsistency Caused by Ancient Sex-Biased Gene Migration

**DOI:** 10.1371/journal.pone.0025549

**Published:** 2011-09-29

**Authors:** Naoki Osada

**Affiliations:** 1 Department of Population Genetics, National Institute of Genetics, Mishima, Japan; 2 Department of Genetics, The Graduate University of Advanced Studies (SOKENDAI), Mishima, Japan; University of Florence, Italy

## Abstract

Inferences of ancient sex-biased migration patterns using sex-linked genetic markers are usually difficult because of a stochastic process of allele fixation. Nevertheless, incongruent phylogenetic trees between different sex-linked markers and between sex-linked and autosomal markers are frequently interpreted as a signature of sex-biased migration without further statistical evaluation. I investigated the types of incongruent phylogenetic trees from which past sex-biased migration events can be statistically supported under the coalescent model. In the case of mammals, detecting a sex-biased migration pattern is not guaranteed by comparing the phylogenetic pattern of mitochondrial and Y-chromosomal loci. Likewise, evidence of introgression at a mitochondrial locus, but not at autosomal loci, does not support the hypothesis of an ancient female-biased migration pattern with statistical significance. In contrast, evidence of introgression at ≥5 unlinked autosomal loci, but not at a Y-chromosomal locus, would reject the null hypothesis of a sexually equal migration rate with statistical significance. A similar argument can be made to infer a male-biased migration pattern. Furthermore, the investigation of many recombining sex-biased markers such as X-chromosomal loci in mammals has the potential to efficiently detect ancient sex-biased demographic patterns.

## Introduction

In sexual organisms, sex-biased migration, in which individuals of one sex are more frequently dispersed than those of the other sex, is widely observed in nature in a wide range of taxa, such as insects, birds, mammals, and plants [1], [2]. Dozens of studies have successfully estimated sex-biased migration rates using sex-specific genetic markers [3]–[7]. The most frequently used sex-linked genetic markers are mitochondrial markers, because mtDNA is abundant in cells and the genome is inherited predominantly through one sex. Moreover, in mammals, the Y-chromosome can be used to measure the dispersal rate of males.

In addition to migration between local populations within the same species, many studies suggest that gene introgression between different species could have occurred not only in bacteria and plants, but in higher animals (reviewed in [8]). Several studies have reported that sex-linked markers showed tree topologies differing from those of other sex-linked and/or autosomal markers among currently reproductively isolated species [9]–[14], and suggested past sex-biased migration events after the initial isolation of species (the migration is often referred to as secondary contact) [10], [12]–[14]. As already known, sex-biased migration is not the only explanation for incongruent genealogies among sex-linked makers. For example, loci responsible for hybrid incompatibility are likely rejected by the recipient genomes and may not introgress to other species [9], [15]. However, explanation by sex-biased migration is often preferred, partly because socio-sexual structures of species are important subjects in ecology and evolution research, and there are several *a priori* assumptions about sex-dependent dispersal rates from observations in fields. For example, Roos et al. found two types of phylogenetic inconsistencies between sex-linked and autosomal markers in colobine monkeys after excluding confounding factors such as tree inference ambiguity and incomplete lineage sorting: introgression at a mitochondrial locus but not at nuclear loci in African colobines, and introgression at nuclear loci but not at a mitochondrial locus in Asian colobines, which may support female- and male-biased migration in African and Asian colobines, respectively [14].

**Figure 1:**
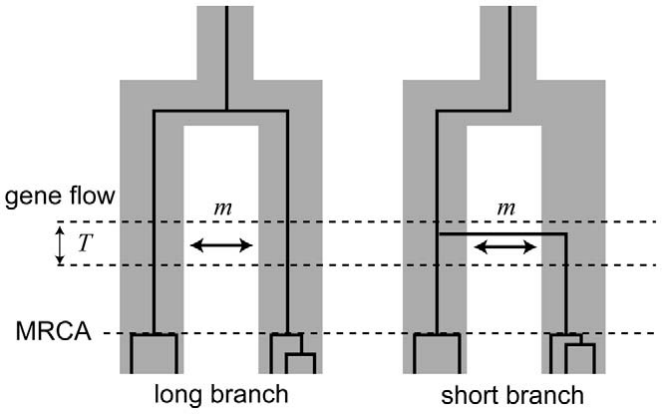
Model of gene flow after speciation. Gene flow between species occurred at rate *m* during time *T* (unit of 2*N* generations) before the occurrence of MRCA. If a migrated gene becomes fixed in the recipient species, a short branch (right panel) is observed; otherwise, long branch is observed (left panel).

When migration is ongoing, it is relatively easy to infer a sex-biased migration pattern by considering the level of genetic admixture between populations at different loci. However, if the migration preceded the occurrence of most recent common ancestors (MRCA) of each species (see Figure 1), we hardly estimate the actual migration rate (the number of migrants per generation) between populations in the past [16]. If the migration rate was small, only a small number of migrant genes could move between populations via hybrid individuals. Those migrant genes, by genetic drift, may completely replace the genes in the recipient population, or may go extinct in the recipient population. In theory, the chance of fixation of migrant genes would be proportional to the number of migrant genes; the fixation process is largely governed by random genetic drift unless migration rate is very high or natural selection acts on the migrant genes. Because most sex-linked loci are recombination-free, it is hard to deal with the stochastic variance in a genealogical inference to conclude the pattern of sex-biased migration. In order to take account of the stochastic variance, we need to test whether the observed data could be obtained simply by genetic drift without sex-biased migration, or could support sex-biased migration with a statistical significance. In the case of Roos et al. [14] the incongruent phylogenetic patterns in primates are supportive evidence for past sex-biased migration events at some extent. However, we wish to quantify the level of confidence in a statistical framework.

In this study, I investigate the types of incongruent phylogenetic trees that could be rigorous evidence of past sex-biased migration events under the coalescent model. We assume the same genetic systems as mammals; males had one X chromosome and one Y chromosome, females had two X chromosomes, and the mitochondrial genome is passed to the next generation only through females. Furthermore, it was assumed that the effective population sizes for males and females are equal. In reality, many factors may lead a sex-biased effective population size even if the census population sizes of males and females are the same (e.g, [17]). For example, polygyny often results in a smaller effective population size at a Y chromosomal locus. Nevertheless, if one wishes to infer sex-specific demography by indirect methods using genetic markers, one should reject the null hypothesis of neutral expectation, i.e., equal demographic patterns between males and females.

## Results

Figure 1 shows a simple model of past secondary contact. In this model, two incipient species with effective population size *N*, have split once and contacted again at a sufficiently later time. Gene flow occurred during time *T*, where time is a unit of 2*N* generations and migration rate between populations (*m*) was assumed to be symmetric. The migration rates of females and males per generation are represented as *m*_F_ and *m*_M_, respectively. Under these assumptions, *N*(*m*_F_+*m*_M_) is the number of migrated autosomes per generation. Likewise, ½*Nm*_F_ and ½*Nm*_M_ mitochondrial and Y-chromosomal genomes are transferred per generation, respectively. A short branch would serve as evidence of gene introgression when the migrant gene had fixed in the population (Figure 1, right panel); otherwise, a long branch would be observed (Figure 1, left panel). For simplicity, it was assumed that the correct genealogies were always inferred from molecular data. Therefore, the method is applicable to reconstructed phylogenetic trees deduced from any type of molecular data, such as nucleotide substitution, microsatellites, and retrotransposon insertion/deletion. The rate of coalescence was approximated using Markov chain iterations for some range of total migration rates (2*Nm*) and fractions of female migrants (*m*_F_/(*m*_F_+*m*_M_)) with sufficiently small intervals. Throughout the following analyses, *T* = 1 was assumed; however, the patterns were consistent for any value of *T* because the product of *m* and *T*, the total number of migrants, is the key parameter.

**Figure 2:**
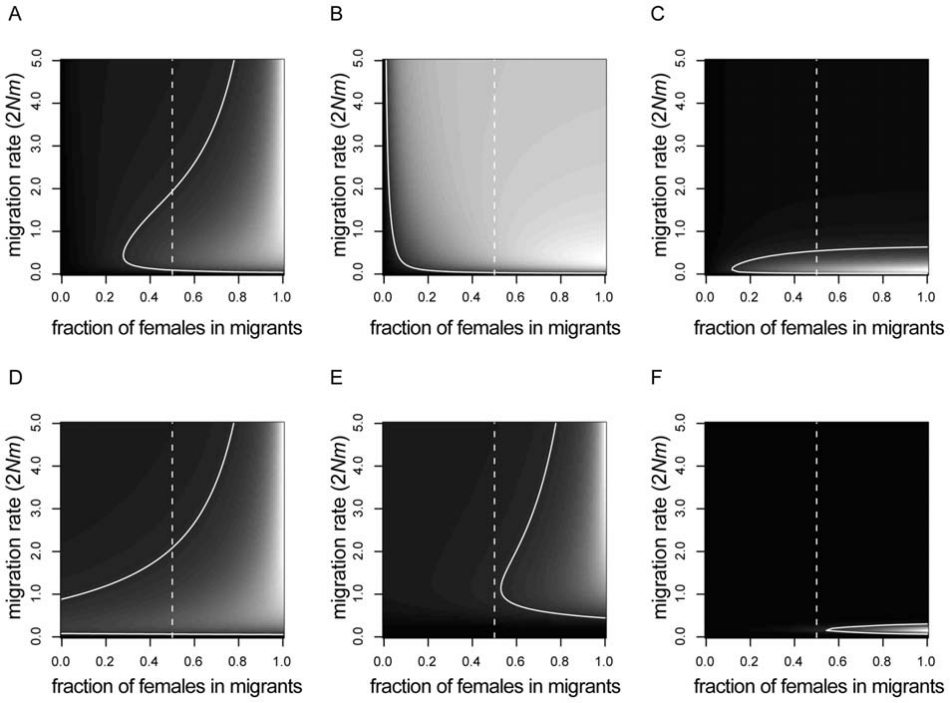
Probability densities of observing various phylogenetic patterns under sex-biased migration rates when T = 1. The abscissa and ordinate axes represent the total migration rate (2*Nm*) and female/male migration rate bias (*m*_F_/(*m*_F_+*m*_M_)). Lighter areas indicate higher probabilities. Dashed and solid lines denote the equal male/female migration rates and 95% CI, respectively. A) Short mitochondrial and long Y-chromosomal branches. B) Short mitochondrial and long autosomal (single locus) branches. C) Short mitochondrial and long autosomal (five unlinked loci) branches. D) Long Y-chromosomal and short autosomal branch (single locus). E) Long Y-chromosomal and short autosomal (five unlinked loci) branches. F) Short X-chromosomal (10 unlinked loci) and long autosomal (30 unlinked loci) branches.

When short and long branches were observed at mitochondrial and Y-chromosomal loci, respectively, we were tempted to conclude that there was female-biased gene flow during the secondary contact. However, this is not always the case. Figure 2A shows the probabilities of observing short mitochondrial and long Y-chromosomal branches with various parameter values. If we assume the parameter values with the highest probability as the maximum likelihood estimators, a confidence interval (CI) can be estimated using the likelihood ratio at each point. The CI was estimated assuming the chi-square distribution with one degree of freedom. The result indicates that the 95% CI of total migration rate and female/male migration ratio is considerably large (the solid lines in Figure 2) and includes the points of female/male migration ratio of 1 (the dashed lines in Figure 2), suggesting that incongruent tree topologies between mitochondrial and Y-chromosomal loci are not sufficient evidence of sex-biased migration.

Similarly, the probabilities of obtaining short mitochondrial branch and long autosomal branches is shown in Figure 2B; it is difficult to prove that the migration was biased toward females. Analyzing multiple autosomal loci would greatly reduce the variance of coalescence time. Assuming that we investigated five unlinked autosomal loci and all loci showed long branches, the 95% CI of the parameters were reduced as shown in Figure 2C. In this case, we can infer that the migration rate was not so high, because high female migration rates are also expected to intermingle the genealogy at autosomal loci. The 95% CI, however, still contains the region of an equal sex migration rate, indicating that we could not reject the null hypothesis. The 95% CI crossed the line of 1∶1 sex migration rate even when 100 autosomal loci took long branches (data not shown). Therefore the biased pattern is not a sufficient condition for sex-biased migration.

Instead of looking at the incongruence at a mitochondrial locus, we may detect the signature of sex-biased migration using a Y-chromosomal locus. The probabilities of observing a short branch at single autosomal locus and a long branch at a Y-chromosomal locus is shown in Figure 2D, in which the 95% CI crossed the line of 1∶1. As shown in Figure 2E, however, when we increase the number of autosomal loci with short branches to five, we are able to reject equal migration rates between sexes at the 5% significance level. In the comparison of phylogenies between Y-chromosomal and autosomal loci, at least five autosomal loci are required for showing the 5% significance level (data not shown).

Summarizing the results using mitochondrial, Y-chromosomal, and autosomal data, inferring the female biased migration rate was not effective by comparing the phylogenetic patterns of mitochondrial and Y-chromosomal loci (Figure 2A). Likewise, evidence of introgression at a mitochondrial locus but not at autosomal loci does not support the hypothesis of past female-biased migration with statistical significance even when multiple autosomal loci were sampled (Figure 2B and 2C). In contrast, evidence of introgression at a sufficient number of autosomal loci but not at a Y-chromosomal locus implies a female-biased migration pattern (Figure 2E). Because mitochondrial and Y-chromosomal loci have equivalent effective population sizes, a male-biased migration pattern could be inferred by swapping mitochondrial and Y-chromosomal data.

In addition to non-recombined sex-linked markers such as mitochondrial and Y-chromosomal loci, recombining sex-linked loci have the potential to detect past sex-biased migration rates [17]–[19]. In case of mammals, X-chromosomal loci are the recombining sex-linked loci. When one female migrates, two X chromosomes move from one population to another, while when one male migrates, only one X chromosome moves. Therefore, a female migration has a twice the effect of a male migration on the X chromosome. Since the X chromosomes recombine during oogenesis, it may contain more information about the inference of sex-biased migration by comparing with the pattern in autosomal loci.

Investigating various X-chromosomal loci helps to reduce the CI of demographic parameters. Figure 2F shows the probabilities of 10 X-chromosomal loci having short branches and 30 autosomal loci having long branches. The pattern rejected the null hypothesis of sexually equal migration rates. Therefore, investigating several X-chromosomal loci has the potential to detect a sex-biased demographic pattern.

## Discussion

Phylogenetic inconsistencies between sex-linked and autosomal loci have been described in many sexual organisms. Although the degree of intensity differs among studies, sex-biased migration is a popular interpretation of such incongruent patterns. However, as shown in this study, phylogenetic incongruence is not a rare event especially when the migration rate during secondary contact was not so high, e.g., 2*Nm*≤1. This range of migration rate is common between recently isolated species, which was estimated using multi-locus DNA data [20]–[23]. Because the rate of gene flow during secondary contact is probably equal to or smaller than the rate between newly emerged species, we should carefully interpret the data of phylogenetic incongruence. Here, I would like to cover a few studies for discussing the confidence in the interpretations of incongruent phylogenies.

Ropiquet and Hassanin [10] identified the pattern of mtDNA introgression in wild goat species, and concluded that the exclusive introgression at mitochondrial locus can be explained by female-biased introgression in the past. Likewise, Nakagome et al. [12] investigated the phylogenetic patterns of six bear species at mitochondrial, Y-chromosomal, and X-chromosomal loci. They observed short and introgressive divergence at Y-chromosomal loci and raised three possibilities for the inconsistency: selective sweep, background selection, and male-biased migration. As shown in this study, exclusive introgression at a mitochondrial or Y-chromosomal locus could happen with relatively high probabilities even under sexually equal migration rate. Therefore, the explanation of random genetic drift after secondary contact should be added to their hypotheses.

Sex-biased dispersal patterns have also been observed in primates. In the case of Asian colobines studied by Roos et al. [14], short branches at many autosomal loci but not at a mitochondrial locus was observed. The pattern statistically rejects the null hypothesis of sexually equal migration rate (see also Fig. 3 of Zinner et al. [7]). In contrast, the evidence of introgression at a mitochondrial locus but not at many autosomal loci in African colobines was not conclusive evidence of a female-biased migration pattern.

Exclusive introgression at a mitochondrial locus has been observed in a wide range of organisms [9]. In these organisms, especially when sex-dependent dispersal patterns have not been known, hypotheses other than female-biased dispersal, such as different hybrid viability and reproductivity between sexes, are often invoked. For example, organisms with heterogametic females such as birds and butterflies, females are often inviable or sterile in inter-specific hybrids, following the Haldane's rule [24]. Although the model is slightly different from those described in this study, the ideas are equivalent assuming a simple hybrid incompatibility model. For example, when a half of female F1 hybrids are completely inviable or sterile, the effective migration rate at mitochondrial and autosomal loci would reduce to ½ and ¾, respectively. The situation is exactly same as the model of 2∶1 migration ratio between males and females described in this study.

Other than the Haldane's rule hypothesis, there are numerous hypotheses explaining the dominance of mtDNA introgression, such as the selection-linkage hypothesis [25] and sexual selection hypothesis [9], [26]. Although it is hard or almost impossible to disentangle these complex factors, we might be able to converge these factors into effective migration rates and make those hypotheses testable using the method described in this study. As shown in the results, a single or few observations of mitochondria-nuclear tree incongruence that occurred before MRCA of sampled populations are not strong evidence of sex-dependent mechanisms; however, if exclusive introgression at a mitochondrial locus is universally observed in some genera or families, the observation might support the prevalence of sex-dependent mechanisms.

In conclusion, incongruent phylogenies between sex-linked makers and between sex-linked and autosomal markers should be carefully interpreted, because a large stochastic variance of genealogies is expected in such markers. It should be noted that models examined in this study are probably too simplified and do not reflect actual demography. In addition, current methods consider only the incongruence of tree topologies. Incorporating more complex demographic scenarios and utilizing more informative data may increase the power of statistical tests and necessary to be developed in future. For example, if the period of gene flow (*T*) is relatively long and a large number of nucleotide sequences across genomes are sequenced, the actual timing of individual migrations could be estimated by measuring the distribution of changes between the nucleotide sequences of two species. Reducing the possible range of migration rate would help to further identify a sex-biased migration pattern. Nevertheless, testing whether a simple sex-biased demographic pattern solely explains the observed incongruence is the first step for investigating more complex scenarios.

## Methods

Past secondary contact scenario with a symmetric migration rate was considered (Figure 1). Suppose that two populations of diploid organisms have an effective population size of *N*. Before the occurrence of MRCA of each population, individuals migrate between populations at rate *m* per generation during time *T* = 2*N* generations. The two lineages can have three types of states: 1) two sampled individuals are coalesced, 2) two sampled individuals are in different populations, and 3) two sampled individuals are in the same population. Let the vector ***G*** *_t_* be the probability of states of an autosomal locus in the *t*-th generation, where the first, second, and third elements represent probabilities of states 1, 2, and 3, respectively. The generation is counted backward in time from the end of secondary contact. The probabilities of two simultaneous migration events and those of simultaneous migration and coalescent events were ignored. The probability of each state at (*t*+1)-th generation is given by the following formula:
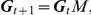


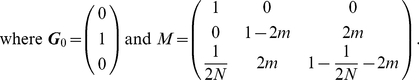
(1)The probability of each state after the secondary contact (*G_T_*) is approximated by iterating the multiplications 2*N* times. The effective population size at mitochondrial and Y-chromosomal loci was assumed to be 1/2*N*, and the effective population size at X-chromosomal loci was assumed to be 3/4*N*. Likewise, migration rate *m* in the equation 1 is replaced to (*m*_F_+*m*_M_)/2, *m*_F_, *m*_M_, and (2*m*_F_+*m*_M_)/3 at autosomal, mitochondrial, Y-chromosomal, and X-chromosomal loci, respectively. The matrix calculations and graphical representations were performed using the R statistical package (http://www.r-project.org/), and the source code is available upon request.
